# Effect of Ultrasonic Nano-Crystal Surface Modification (UNSM) on the Passivation Behavior of Aged 316L Stainless Steel

**DOI:** 10.3390/ma10070713

**Published:** 2017-06-27

**Authors:** Ki-Tae Kim, Jung-Hee Lee, Young-Sik Kim

**Affiliations:** Research Center for Energy and Clean Technology, School of Materials Science and Engineering, Andong National University, 1375 Gyeongdong-ro, Andong, Gyeongbuk 36729, Korea; kitae1@pyunji.andong.ac.kr (K.-T.K.); jhlee@pyunji.andong.ac.kr (J.-H.L.)

**Keywords:** 316L stainless steel, aging, UNSM, passivation, sensitization, carbon segregation

## Abstract

Stainless steels have good corrosion resistance in many environments but welding or aging can decrease their resistance. This work focused on the effect of aging time and ultrasonic nano-crystal surface modification on the passivation behavior of 316L stainless steel. In the case of slightly sensitized 316L stainless steel, increasing the aging time drastically decreased the pitting potential, increased the passive current density, and decreased the resistance of the passive film, even though aging did not form chromium carbide and a chromium depletion zone. This behavior is due to the micro-galvanic corrosion between the matrix and carbon segregated area, and this shows the importance of carbon segregation in grain boundaries to the pitting corrosion resistance of stainless steel, in addition to the formation of the chromium depletion zone. UNSM (Ultrasonic Nano Crystal Surface Modification)-treatment to the slightly sensitized 316L stainless steel increased the pitting potential, decreased the passive current density, and increased the resistance of the passive film. However, in the case of heavily sensitized 316L stainless steel, UNSM-treatment decreased the pitting potential, increased the passive current density, and decreased the resistance of the passive film. This behavior is due to the dual effects of the UNSM-treatment. That is, the UNSM-treatment reduced the carbon segregation, regardless of whether the stainless steel 316L was slightly or heavily sensitized. However, since this treatment made mechanical flaws in the outer surface in the case of the heavily sensitized stainless steel, UNSM-treatment may eliminate chromium carbide, and this flaw can be a pitting initiation site, and therefore decrease the pitting corrosion resistance.

## 1. Introduction

Since austenitic stainless steels have good properties of strength and toughness, and high corrosion resistance, they have been widely used in the field of power plants, chemical plants, and other industries [1]. The high corrosion resistance of austenitic stainless steel is due to the chemical stability of the passive film formed on the surface, and the properties of the film depends upon the alloying elements of the steels. Basically, stainless steel can be defined as having a chromium content of over 12%; thus the passive film is mainly composed of Cr_2_O_3_, and the corrosion resistance can be improved by the addition of Ni, Mo, W, N, etc.

However, in many environments, austenitic stainless steels are sensitive to intergranular corrosion, pitting, and stress corrosion cracking [2,3]. In particular, if the steel may be slowly cooled or exposed over the range of sensitization temperatures (500–850 °C) for a long time after the solution heat treatment at over 1050 °C, intergranular chromium carbide may be formed and may then reduce the corrosion resistance [1]. Generally, chromium carbide is very stable under 850 °C, the chromium diffusion rate forming the carbide over 500 °C is very fast, and the stainless steel can then be thermodynamically sensitized in this temperature range [4,5]. Therefore, grain boundaries result in chromium carbide precipitation. If the sensitized stainless steel is exposed to corrosive environments, the chromium depletion zone will preferentially corrode [6,7]. On the basis of the time-temperature-precipitation curve, carbide can precipitate in the range of 500–800 °C, depending upon the sensitization time and the alloying system [7]. The chromium carbide Cr_23_C_6_ is the only precipitate that causes chromium depletion which is mainly responsible for the intergranular corrosion of 316L stainless steel [8,9,10,11,12]. Several mechanisms have been explored to explain the dynamics of sensitization, but the chromium depletion theory has been the only one that has been proven experimentally [13]. Increasing the time of sensitization increased the enrichment of Cr_23_C_6_ in stainless steels. This enrichment caused the lowering in nobility and nucleation of the pitting corrosion resistance behaviour of AISI 304 stainless steel [14]. Recently, Kim, et al. [15] newly reported that the intergranular corrosion occurring in both the stabilized ferritic and austenitic stainless steels is induced by Cr-depletion due to segregation of the un-reacted Cr atoms around the carbides of stabilizer elements along the grain boundary, but is not due to the formation of Cr-carbide. The welding process induces the sensitization and the residual stress and can facilitate the corrosion of the steels [16,17]. The welded area can be divided into three regions of weldment, heat-affected zone, and base metal depending upon the microstructures. Among these, the heat-affected zone can be sensitized by the precipitation of chromium carbides and is prone to corrosion such as stress corrosion cracking, intergranular corrosion, and pitting [18].

On the other hand, compressive stress-applied technology such as a laser peening has widely emerged, and is used to enhance the resistance to stress corrosion cracking of austenitic stainless steel [19,20]. It was already proven that after laser peening, the surface properties were enhanced [21]. This technology reduced the surface inclusion and suppressed the pit initiation and improved the pitting resistance by applying compressive stress [22]. In other words, surface treatment can convert the residual stress from tensile stress to compressive stress. Applicable and studied techniques to induce compressive residual stress on the weldments can be summarized as shot peening [23,24,25], laser shock peening [26,27], water jet peening [28], ultrasonic peening [29], and UNSM (Ultrasonic Nano-crystal Surface Modification) [30,31]. In a typical UNSM technique, a tungsten carbide (WC) tip is attached to an ultrasonic horn, which strikes the specimen surface at up to 20,000 or more times per second, with 1000 to 10,000 shots per square millimeter in a very short time. These strikes, which can be described as micro cold forging, bring severe plastic deformation to the surface layers, and thus induce the nano-crystalline structure [30]. The nano-crystalline surface could increase the density of diffusion paths available for alloying elements to migrate and rapidly form a protective passive film [32,33]. In addition, residual stress in the surface layer affects the critical current density and the current density for passivation, which are closely related to the formation and retention of the passivation layer [34]. The high mechanical energy imparted by UNSM to the steel surface may also induce the diffusion of the alloying elements [30,31,33], and can therefore affect the intergranular corrosion resistance of the stainless steels.

The C-curves of the Time-Temperature-Sensitization (TTS) diagram of AISI 316 stainless steel are displaced towards lesser time by increasing the cold work, and sensitization of the AISI 316 stainless steel accelerated the precipitation of the Cr_23_C_6_ carbide [35]. Remarkable improvements in the Degree of Sensitization (DOS) and the Intergranular Corrosion (IGC) of type 304 (UNS S 30400) and type 316L (UNS S 31603) stainless steels were achieved through extreme grain boundary randomization [36]. Recently, our group reported that even though chromium carbide was not precipitated, aging time drastically increased the intergranular corrosion rate of 316L stainless steel, and we confirmed that the increased intergranular corrosion rate of slightly sensitized (not carbide formed) 316L stainless steel was due to the carbon segregation along the grain boundaries. UNSM treatment improved the intergranular corrosion resistance of aged stainless steels, and the improvement was due to the reduction of carbon segregation and the grain refinement of the outer surface, including the introduction of compressive residual stress [5].

As discussed above, the UNSM treatment improves the mechanical properties and induces the nano-crystallization of the outer surface, but there has been little research on the effects of UNSM on the anodic polarization behavior of stainless steel. This work, therefore, focused on the effect of UNSM treatment on the anodic polarization behavior of aged 316L stainless steel and proposed its mechanism.

## 2. Materials and Methods

### 2.1 Specimen

Table 1 shows the chemical composition of the AISI 316L austenitic stainless steel used in this work. Aging treatments were performed at 650 °C for 1, 5, and 48 h in N_2_ protective atmosphere, and then the steel was water quenched. UNSM treatment was applied to the surface using UNSM equipment (Design Mecha-LM20 UNSM system, Asan, Korea). Table 2 shows the UNSM treatment conditions.

**Figure 1:**
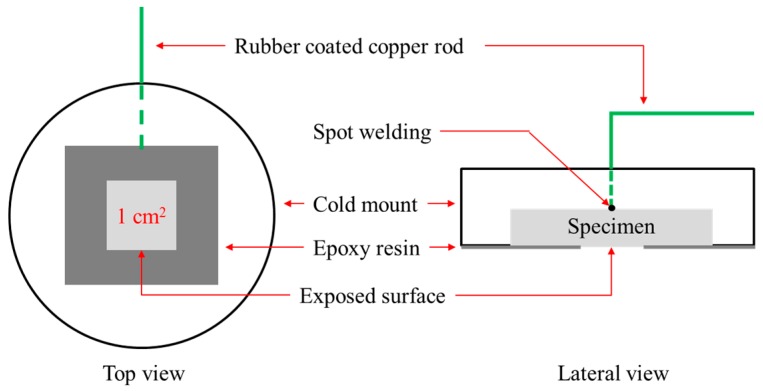
Shape and dimensions of the electrochemical test specimen.

Figure 1 shows the shape and dimensions of the specimen for the electrochemical test (anodic polarization, AC-impedance, and Mott-Schottky tests).

### 2.2. Corrosion Test

#### 2.2.1. Anodic Polarization Test

Specimens were cut to a size of 1.5 × 1.5 mm, and after electrical connection, they were epoxy-mounted, and the surface was ground using #600 SiC paper and coated with epoxy resin, except for an area of 1 cm^2^. The anodic polarization test was performed in 3.5% NaCl solution using a potentiostat (Gamry DC 105, Warminster, VA, USA) and the reference electrode was a saturated calomel electrode (SCE), and the counter electrode was high-density graphite rods. The test solution was de-aerated using nitrogen gas at the rate of 100 mL/min for 30 min and the scanning rate was 0.33 mV/s. The pitting potential that is the least positive potential at which pits can form was determined from the anodic polarization curve.

#### 2.2.2. AC Impedance Measurement

In order to measure the AC impedance, the specimens were ground using #2000 SiC paper, and then polished using a diamond paste (3 μm). The test solution was the same as that of the polarization test. The AC impedance measurement was performed using an electrochemical analyzer (Gamry EIS 300, Warminster, VA, USA). Before measuring, passivation was treated at +0 mV (SCE) for 30 min. AC impedance was measured at a passivation potential from 10 kHz to 0.01 Hz and the AC voltage amplitude was 10 mV.

#### 2.2.3. Mott-Schottky Test

The Mott-Schottky plot was prepared to determine the semiconductive properties of the passive film. The specimen preparation was the same as that for the AC impedance measurement and the DC amplitude was 10 mV (peak-to-peak) at 1580 Hz of the AC frequency [37]. The capacitance was measured at the scan rate of 50 mV/s from +0.5 V (SCE) to −1 V (SCE).

### 2.3. Microstructure Analysis

The specimen was cut to a size of 20 × 20 × 5 mm and then ground with #2000 SiC paper and polished with a 3 µm diamond paste. Finally, the specimen was cleaned with ethyl alcohol, using an ultrasonic cleaner. The microstructure was observed by optical microscopy (AXIOTECH 100HD, ZEISS, Oberkochen, Germany) and SEM-EDS (VEGA II LMU, Tescan, Brno, Czech Republic), after etching by aqua regia (70 mL HNO_3_ + 30 mL HCl). Also, an Electron Probe Micro Analyzer (EPMA-1600 15 kV, Shimadzu, Kyoto, Japan) was used to identify the elemental distribution of the passivated surface.

## 3. Results

### 3.1. Effect of Aging Treatment on the Electrochemical Passivation Behavior

**Figure 2:**
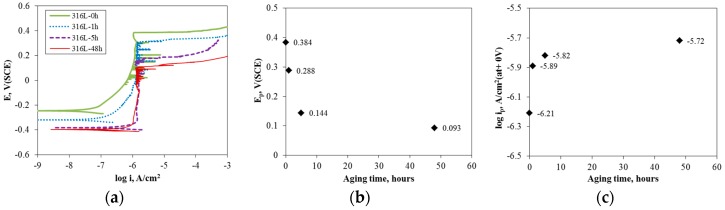
Effect of aging time at 650 °C on the polarization behavior of 316L stainless steel (de-aerated 30 °C, 3.5% NaCl): (**a**) anodic polarization curves; (**b**) pitting potential with aging time; and (**c**) passive current density with aging time.

Figure 2 shows the effect of aging time at 650 °C on the polarization behavior of the 316L stainless steel in de-aerated 3.5% NaCl at 30 °C. In the case of the annealed specimen (316L-0 h), the corrosion potential and pitting potential were high, but the aging treatment degraded the anodic polarization behavior of the 316L stainless steel. Increasing the aging time lowered the pitting potential and also increased the passive current density at 0 V (SCE). Generally, the aging treatment formed chromium carbides in the grain boundary and grain, and then generated a chromium-depleted zone near the carbide. Therefore, pitting corrosion was easily initiated at this chromium-depleted zone, and the pitting resistance was reduced. However, it is interesting to note that the pitting potential of even the 1 or 5 h-aged specimen decreased drastically, and their passive current densities greatly increased. From a thermodynamical aspect [8], short aging times such as 1 or 5 h could not form chromium carbides, but did degrade their passivation behavior. A similar pattern of corrosion by aging time was already observed in the intergranular corrosion behavior [4,38].

**Figure 3:**
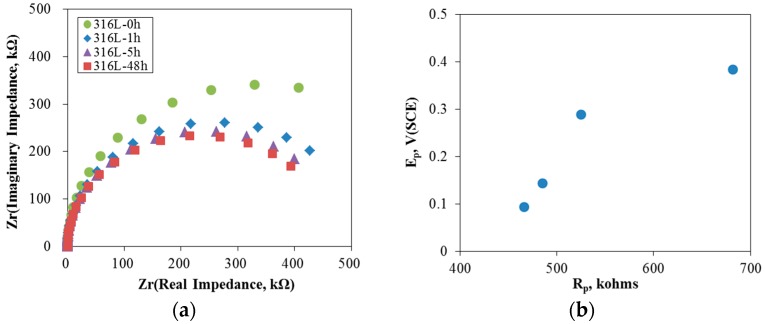
AC impedance measurement on 316L stainless steel at 0 V (SCE) in de-aerated 30 °C, 3.5% NaCl: (**a**) Nyquist plot; and (**b**) R_p_ vs. pitting potential.

Figure 3 shows the results for the AC impedance measurement on 316L stainless steel at 0 V (SCE) in de-aerated 30 °C, 3.5% NaCl solution. The passive film was formed at 0 V (SCE) for 30 min and the AC impedance was measured. Figure 3a shows that increasing the aging time depressed the Nyquist plots and their impedance circles. The polarization resistance was calculated using a Randles model. Figure 3b compares the relationship between the polarization and pitting potential. The figures show that increasing the polarization resistance of stainless steels increases the pitting potential. That is, it should be noted that the properties of a passive film are important for enhancing the resistance to pitting corrosion.

**Figure 4:**
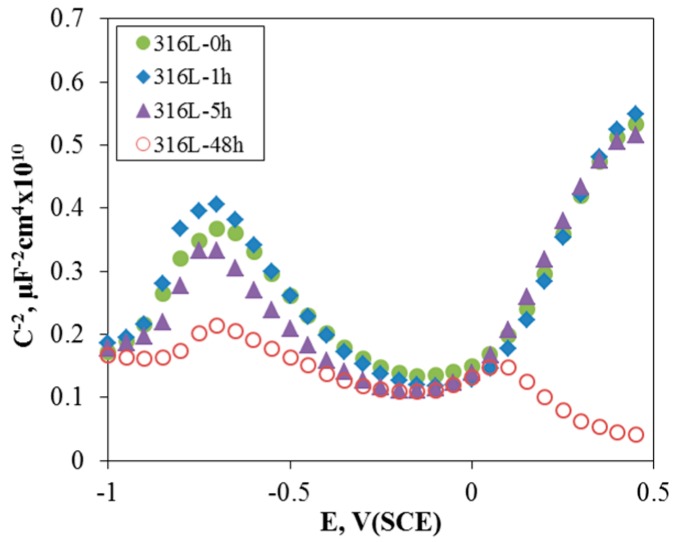
Effect of aging time on the Mott-Schottky plot of the passive film formed at 0 V (SCE) for 30 min in de-aerated 3.5% NaCl solution at 30 °C.

Figure 4 shows the effect of aging time on the Mott-Schottky plot of the passive film formed at 0 V (SCE) for 30 min in de-aerated 3.5% NaCl solution at 30 °C. Through the Mott-Schottky plot, we can find the bipolarity [39,40,41] of the passive film, and the concentration of donors and acceptors by the point defect model for a passive film [42,43]. In this Mott-Schottky plot, a positive slope means n-type semiconductive properties, while a negative slope implies p-type semiconductive properties. Therefore, the passive film formed on 316L stainless steel in sodium chloride solution reveals p-n type semiconductive properties, i.e., the bipolar film. Increasing the aging time still did not change the n-type properties, but did gradually reduce the slope of the p-type properties. In other words, the aging treatment to stainless steel deteriorated the bipolar properties, especially the p-type property and therefore reduced the pitting corrosion resistance.

### 3.2. Effect of UNSM on the Electrochemical Passivation Behavior

**Figure 5:**
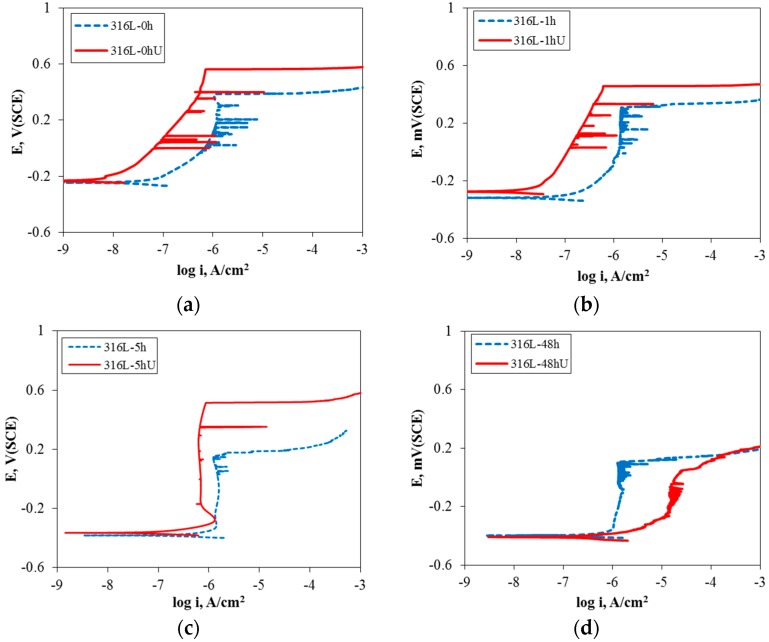
Effect of UNSM on the polarization behavior of 316L stainless steel aged at 650 °C in de-aerated 30 °C, 3.5% NaCl solution; (**a**) non-aged specimen; (**b**) 1 h-aged specimen; (**c**) 5 h-aged specimen; and (**d**) 48 h-aged specimen.

**Figure 6:**
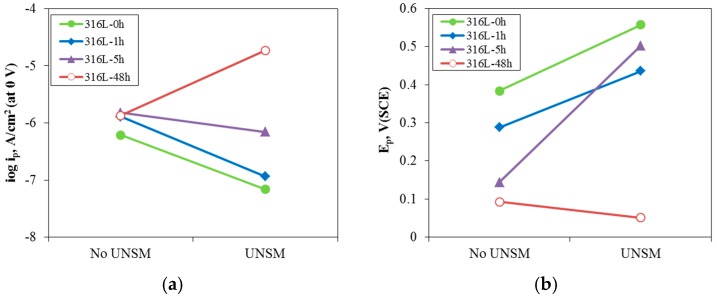
Effect of UNSM on (**a**) passive current density; and (**b**) pitting potential obtained in de-aerated 3.5% NaCl solution at 30 °C.

Figure 5 shows the effect of UNSM on the polarization behavior of 316L stainless steel aged at 650 °C in de-aerated 3.5% NaCl solution at 30 °C. In the case of the non-aged specimen, UNSM-treatment (316L-0hU) improved the anodic polarization behavior. In contrast, the UNSM-treatment increased the anodic polarization behavior of 1 h and 5 h-aged specimens, but the treatment degraded the behavior of the 48 h-aged specimen. Figure 6 reveals the effect of UNSM on the passive current density and pitting potential obtained in de-aerated 3.5% NaCl solution at 30 °C. In summary, the UNSM-treatment on the slightly sensitized specimen reduced the passive current density and increased the pitting potential, while the treatment on the heavily sensitized specimen increased the passive current density and lowered the pitting potential. It is interesting that the UNSM-treatment enhanced the intergranular corrosion in even the heavily sensitized specimen, in addition to the slightly sensitized specimen [4,38].

**Figure 7:**
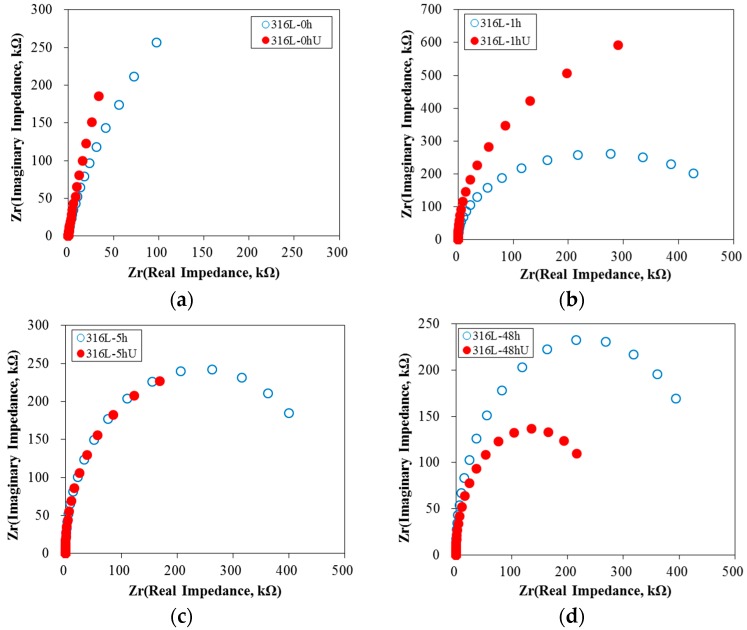
Effect of UNSM on the Nyquist plot of 316L stainless steel at 0 V (SCE) in de-aerated 30 °C, 3.5% NaCl solution: (**a**) non-aged specimen; (**b**) 1 h-aged specimen; (**c**) 5 h-aged specimen; and (**d**) 48 h-aged specimen.

**Figure 8:**
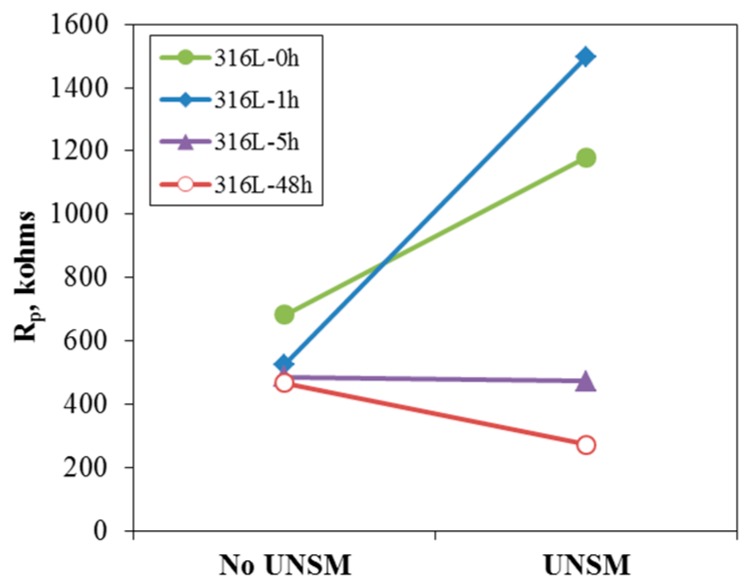
Effect of UNSM on the resistance of the passive film of 316L stainless steel at 0 V (SCE) in de-aerated 30 °C, 3.5% NaCl solution.

**Figure 9:**
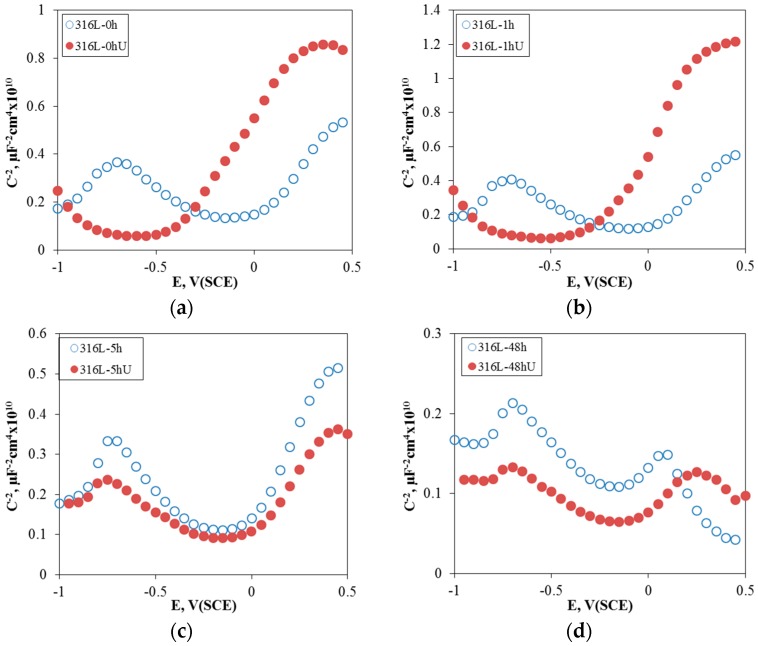
Effect of UNSM on the Mott-Schottky plot of the passive film formed at 0 V (SCE) for 30 min. in de-aerated 30 °C, 3.5% NaCl solution: (**a**) non-aged specimen; (**b**) 1 h-aged specimen; (**c**) 5 h-aged specimen, and (**d**) 48 h-aged specimen.

Figure 7 shows the effect of UNSM on the Nyquist plot of 316L stainless steel at 0 V (SCE) in de-aerated 30 °C, 3.5% NaCl solution. Except for the 48 h-aged specimen, the Nyquist plots of non- or slightly-aged specimens were improved. A Randles model was used to calculate the resistances of the passive film of 316L stainless steel at 0 V (SCE) in de-aerated 30 °C, 3.5% NaCl. Figure 8 summarizes the effect of UNSM on the resistance of the passive film. The UNSM-treatment increased the resistances of the passive film of non- or slightly-aged specimens, but the treatment lowered the resistance of the passive film of the heavily sensitized specimen. Figure 9 shows the effect of UNSM on the Mott-Schottky plot of the passive film formed at 0 V (SCE) for 30 min in de-aerated 30 °C, 3.5% NaCl solution. Regardless of the UNSM treatment, all of the specimens revealed p-n type semiconductive properties. The n-type properties (positive slope) of the non- or slightly sensitized specimens were relatively strong, but the n-type properties of the heavily sensitized specimen were relatively weak.

## 4. Discussion

Pitting corrosion initiates by nonmetallic inclusions or second-phase intermetallics in the stainless steel surface microstructure [44]. Stainless steel can be sensitized by aging treatment, and thus chromium carbides and a chromium-depleted zone can be formed near the grain boundaries [45]; and in addition, it was recently reported that carbon segregated areas near grain boundaries of even slightly sensitized stainless steel were formed, and this carbon segregation facilitated the intergranular corrosion [4].

**Figure 10:**
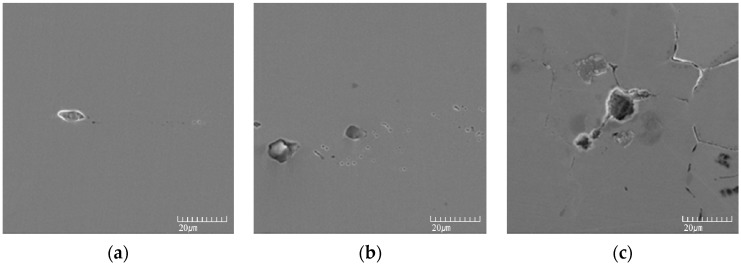
SEM image after the polarization test in de-aerated 30 °C, 3.5% NaCl: (**a**) 1 h-aged; (**b**) 5 h-aged; and (**c**) 48 h-aged.

Figure 2 shows that the pitting potential was greatly reduced in even the slightly sensitized specimen. Figure 10 shows the results of observation of the surface after the anodic polarization test in order to elucidate this corrosion behavior. Figure 10a,b show that pits initiated along many lines and propagated with increasing aging time; while Figure 10c shows that in the 48 h-aged specimen, the corroded area finally looks like the appearance corroded along grain boundaries.

**Figure 11:**
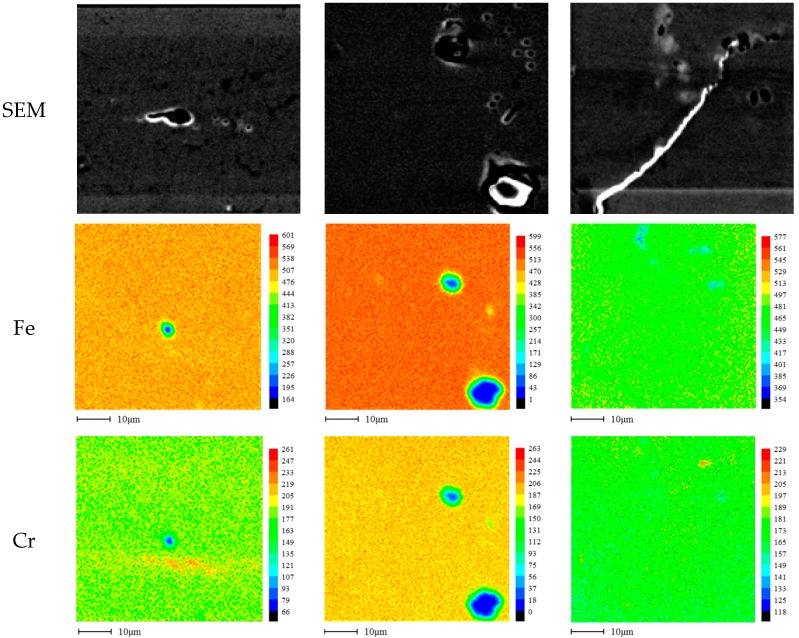

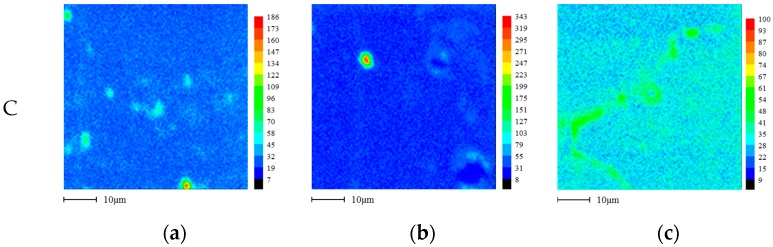
Elemental distribution analyzed by EPMA (Electron Probe Micro Analyzer) on the surface of aged 316L stainless steel after the polarization test in de-aerated 30 °C, 3.5% NaCl: (**a**) 1 h-aged; (**b**) 5 h-aged; and (**c**) 48 h-aged.

Figure 11 shows the EPMA analysis that was therefore performed in order to compare the corroded appearance to elemental correlation. Figure 11 shows the elemental distribution analyzed by EPMA on the surface of the aged 316L stainless steel after the polarization test in de-aerated 3.5% NaCl at 30 °C. In the case of 1 h and 5 h-aged specimens, carbon was relatively more segregated at the corroded area, and carbon segregation can be definitely confirmed along the grain boundaries of the chromium carbide formed specimen (48 h-aged). The passivation behavior of the slightly sensitized stainless steel can be affected by carbon segregation, and the segregated carbon induced micro-galvanic corrosion, which initiated pitting and degraded passivation [13]. After the exposure beyond the critical duration for carbide formation, chromium depletion and carbide formed areas formed, and since these areas are sensitive to pitting initiation, they degraded the passivation.

**Figure 12:**
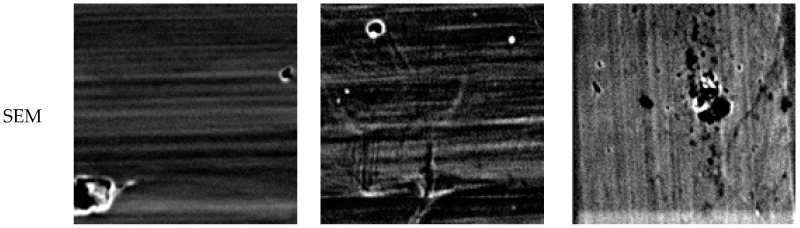

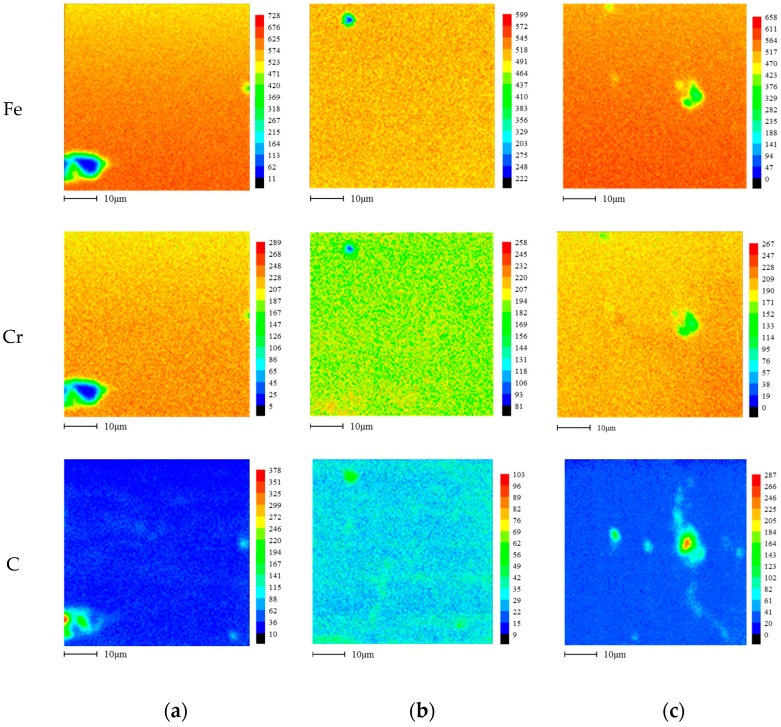
Elemental distribution analyzed by EPMA on the surface of UNSM treated 316L stainless steel after the polarization test in de-aerated 30 °C, 3.5% NaCl: (**a**) 1 h-aged; (**b**) 5 h-aged; and (**c**) 48 h-aged.

**Figure 13:**
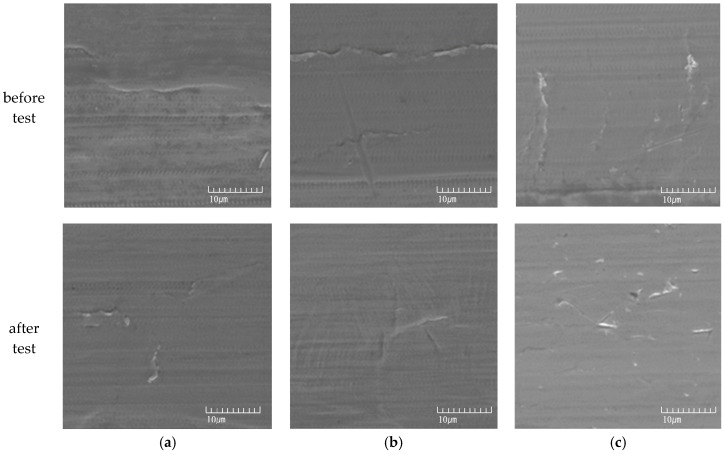
SEM images of UNSM-treated specimens before/after the anodic polarization test in de-aerated 30 °C, 3.5% NaCl: (**a**) 1 h-aged; (**b**) 5 h-aged; and (**c**) 48 h-aged.

On the other hand, the UNSM-treatment improved the passivation of 1 h and 5 h-aged specimens, but decreased the pitting resistance of the 48 h-aged specimen. Wang, et al. [32,33] reported that the nano-crystalline surface could increase the density of diffusion paths available for alloying elements to migrate and rapidly form a protective passive film. According to the results by Takakuwa, et al. [34], residual stress in the surface layer with different surface finishes affects the critical current density and the current density for passivation, the factors of which are closely related to the formation and retention of the passivation layer. Many researchers [30,31,33] reported that the high mechanical energy imparted by various methods to the steel surface may also induce the diffusion of the alloying elements and therefore can affect the intergranular corrosion resistance of the stainless steels. We already proposed that the UNSM treatment improved the intergranular corrosion resistance of the aged stainless steels, and the improvement was due to the reduction of carbon segregation and the grain refinement of the outer surface, including the introduction of compressive residual stress [4]. Figure 12 shows the elemental distribution analyzed by EPMA on the surface of UNSM treated 316L stainless steel after the polarization test in de-aerated 3.5% NaCl at 30 °C. It can be confirmed that the UNSM-treatment reduced carbon segregation regardless of the aging time. As described above, carbon segregation facilitates micro-galvanic corrosion, and therefore the reduction of carbon segregation by the UNSM-treatment should improve the pitting corrosion resistance. In the case of the slightly sensitized specimen, this was true, but the pitting corrosion resistance was decreased in the heavily sensitized specimen in spite of the reduction of carbon segregation by the UNSM-treatment. That is, this behavior requires another explanation. Figure 13 shows SEM images of UNSM-treated specimens before/after the anodic polarization test in de-aerated 3.5% NaCl at 30 °C. Mechanical flaws by the UNSM-treatment can be observed even in the specimen before the anodic polarization test. In particular, in the case of the 48 h-aged specimen, several mechanical flaws were observed; and after the anodic polarization test, it was confirmed that the pitting corrosion initiated and propagated at the flaws. In other words, it should be noted that while the UNSM-treatment can reduce the carbon segregation by aging, it can increase the irregularity of the surface due to mechanical flaws.

**Figure 14:**
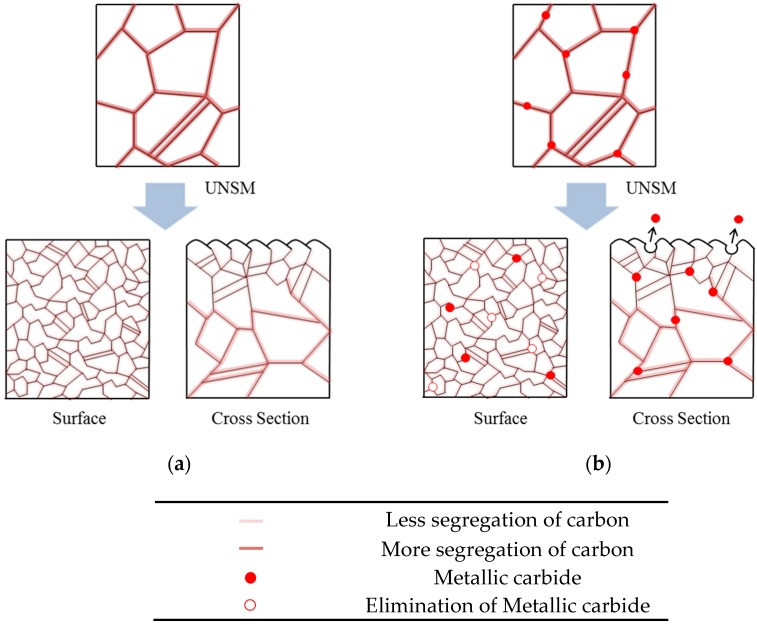
Pitting corrosion model of aged 316L stainless steel by UNSM treatment: (**a**) slightly-aged specimen; and (**b**) over-aged specimen.

Therefore, we propose the pitting corrosion model of the stainless steel by UNSM-treatment. Figure 14 shows the pitting corrosion model of aged 316L stainless steel by the UNSM treatment. In the slightly aged specimen, the UNSM-treatment refines the grain of the outer surface [4,38] and reduces the carbon segregation, and therefore increases the pitting corrosion resistance even though it creates mechanical flaws. On the other hand, in the case of the over-aged specimen, the UNSM-treatment refines the grain of the outer surface, and reduces the carbon segregation similar to the slightly aged specimen, but creates mechanical flaws on the surface, and then these flaws act as the initiation sites of pitting corrosion, and finally decrease the pitting corrosion resistance.

## 5. Conclusions

(1) In the case of slightly sensitized 316L stainless steel, the aging time drastically decreased the pitting potential, increased the passive current density, and lowered the resistance of the passive film, even though chromium carbide and the chromium depletion zone were not formed by aging. This behavior is due to the micro-galvanic corrosion between the matrix and the carbon segregated area, and shows the importance of carbon segregation in grain boundaries to the pitting corrosion resistance of stainless steel, in addition to the formation of the chromium depletion zone.
(2) UNSM-treatment of the slightly sensitized 316L stainless steel increased the pitting potential, decreased the passive current density, and increased the resistance of the passive film. However, in the case of the heavily sensitized 316L stainless steel, the UNSM-treatment decreased the pitting potential, increased the passive current density, and decreased the resistance of the passive film. This behavior is due to the dual effects by UNSM-treatment. That is, UNSM-treatment reduced the carbon segregation regardless of whether the stainless steel 316L was slightly or heavily sensitized. However, since this treatment created mechanical flaws in the outer surface, in the case of the heavily sensitized stainless steel, the UNSM-treatment may eliminate chromium carbide, and this flaw can be a potential pitting initiation site, and therefore decrease the pitting corrosion resistance.

## Figures and Tables

**Table 1:** Chemical composition of commercial 316L stainless steel (wt %).

**Cr**	**Mo**	**Ni**	**C**	**Mn**	**Si**	**P**	**S**	**Fe**
16.69	1.99	10.19	0.01	1.19	0.59	0.04	0.01	Bal

**Table 2:** Conditions of UNSM (Ultrasonic Nano Crystal Surface Modification) treatment on 316L stainless steel.

**Alloys**	**316L Stainless Steel**
Amplitude	30 μm
Static load	10 N
Pitch	0.07 mm
Speed	1000 mm/min
Tip diameter	2.38 mm (WC ^1^)

^1.^ WC : Tungsten Carbide.
